# Fetal Renal Echogenicity Associated with Maternal Focal Segmental Glomerulosclerosis: The Effect of Transplacental Transmission of Permeability Factor suPAR

**DOI:** 10.3390/jcm7100324

**Published:** 2018-10-04

**Authors:** Shirley Shuster, Ghada Ankawi, Christoph Licht, Jochen Reiser, Xuexiang Wang, Changli Wei, David Chitayat, Michelle Hladunewich

**Affiliations:** 1 The Prenatal Diagnosis and Medical Genetics Program, Department of Obstetrics and Gynecology, Mount Sinai Hospital, University of Toronto, Toronto, ON M5G 1Z5, Canada; sshus068@uottawa.ca (S.S.); David.Chitayat@sinaihealthsystem.ca (D.C.); 2 Department of Medicine, Division of Nephrology, Sunnybrook Science Health Sciences Centre, University of Toronto, Toronto, ON M4N 3M5, Canada; ghadaankawi@gmail.com; 3 Division of Nephrology, The Hospital for Sick Children, Toronto, ON M5G 1X8, Canada;christoph.licht@sickkids.ca; 4 Department of Pediatrics, University of Toronto, Toronto, ON M5G 1X8, Canada; 5 Department of Medicine, Rush University Medical Center, Chicago, IL 60612, USA; jochen_reiser@rush.edu (J.R.); Xuexiang_Wang@rush.edu (X.W.); Changli_Wei@rush.edu (C.W.); 6 Division of Clinical and Metabolic Genetics, The Hospital for Sick Children, University of Toronto, Toronto, ON M5G 1X8, Canada

**Keywords:** echogenic kidneys, oligohydramnios, nephrotic syndrome, focal segmental glomerulosclerosis (FSGS), soluble urokinase-type plasminogen activator receptor (suPAR)

## Abstract

We report a case of a pregnant woman with nephrotic syndrome due to biopsy-proven focal segmental glomerulosclerosis (FSGS) whose fetus developed echogenic kidneys and severe oligohydramnios by 27 weeks of gestation. Maternal treatment with prednisone resulted in normalization of the amniotic fluid indices and resolution of fetal renal echogenicity. The newborn was noted to have transient renal dysfunction and proteinuria, resolving by 6 weeks postpartum. The transplacental passage of permeability factors is postulated to have caused both the fetal and newborn renal presentation, with significantly elevated levels of soluble urokinase-type plasminogen activator receptor (suPAR) noted in the cord blood. This case documents the transplacental maternal-fetal transmission of suPAR, demonstrating the potential for maternal-fetal transmission of deleterious, disease-causing entities, and adds to the differential diagnosis of fetal echogenic kidneys. Further, this is the first documentation of a fetal response to maternal systemic therapy.

## 1. Introduction

The pathophysiology of focal segmental glomerulosclerosis (FSGS) is not fully understood. FSGS is a disease that primarily affects the glomerular podocytes. Numerous genetic causes have been identified as disease-causing [1]. In mutation-negative cases, however, the presence of a circulating factor has been postulated. Evidence supporting such a circulating factor, presumably an inflammatory mediated cytokine, is derived from studies that have demonstrated prompt recurrence of the nephrotic syndrome after renal transplantation, as well as studies that have demonstrated that serum from patients with FSGS can increase glomerular permeability to albumin [2,3,4]. The precise identity of the circulating permeability factor remains to be fully established, but recent insights into podocyte biology have identified a urokinase receptor (uPAR) to be integral to the maintenance of the slit diaphragm through its ability to form signaling complexes with other transmembrane proteins, including lipid-dependent activation of αvβ3 integrin [5]. Activation of this receptor and its downstream pathways may result in podocyte effacement, proteinuria, glomerular damage, and impaired renal function. uPAR is a glycosyl-phosphatidylinisotol (GPI)-anchored three-domain (DI, DII, and DIII, as numbered from the *N* terminus) protein, which can be released from the plasma membrane as a soluble molecule, soluble urokinase-type plasminogen activator receptor (suPAR), by cleavage of the GPI anchor [6]. suPAR has been demonstrated to be elevated in patients with FSGS [6], and higher levels accelerate decline of renal function [7,8].

Little is known about FSGS in pregnancy in general, and even less is known about the potential for transplacental transmission of permeability factors to the fetus. In 2001, Kemper et al. [9] described a case in which a mother with FSGS gave birth to a girl who was noted to have proteinuria at delivery that resolved completely shortly thereafter. At that time, it was speculated that a glomerular permeability factor was transmitted from the mother to her child, and blood was archived for future analysis should the circulating FSGS factor be identified. In 2013, Reiser et al. [10] analyzed the original blood samples and found elevated suPAR levels in both the mother (4635 pg/mL) and the newborn (5225 pg/mL), suggesting that suPAR was transmitted from the mother to her newborn. The effect on the newborn was transient as long as the mother transferred suPAR to her. After delivery, suPAR was likely eliminated by the baby’s otherwise normal kidneys and proteinuria resolved.

Here we report a pregnant woman with nephrotic syndrome due to FSGS whose fetus developed echogenic kidneys and oligohydramnios by 27 weeks of gestation and postnatally was noted to have proteinuria with decreased renal function. The disappearance of the fetal findings coincided with maternal treatment with prednisone, and nephrosis in the newborn was transient, resolving by 6 weeks of age. To better understand the case, we also infused human HIS-tagged suPAR into pregnant mice and analyzed the fetal presence of suPAR. The woman provided consent to share her story and the animal studies were approved by the institutional review board at Rush University Medical Center.

## 2. Patient Report

The mother was a 28-year-old G2P0SA1 and the father was 36 years old. The father was healthy and the couple was nonconsanguineous. Their family histories were noncontributory. Her pregnancy was initially uncomplicated, and there was no history of infection or exposure to teratogens. Screening for aneuploidy was unremarkable, and detailed fetal ultrasound done at 21 weeks of gestation showed no fetal abnormalities and normal fetal kidneys.

**Figure 1:**
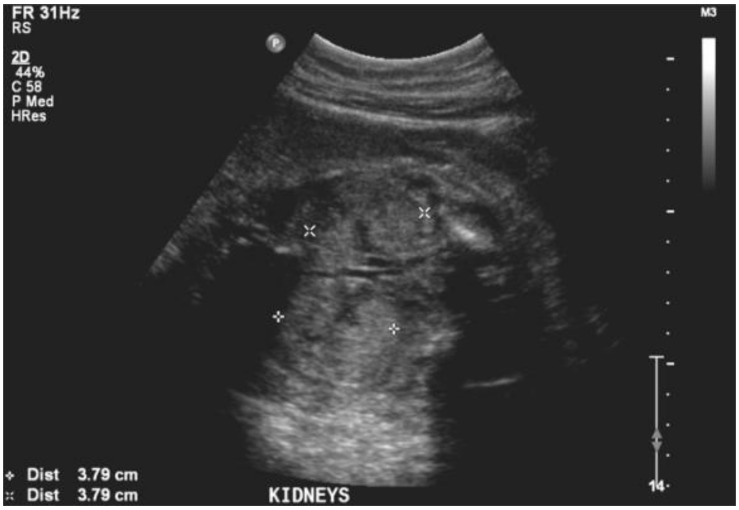
Ultrasound image of fetal kidneys at 27 weeks of gestation.

**Figure 2:**
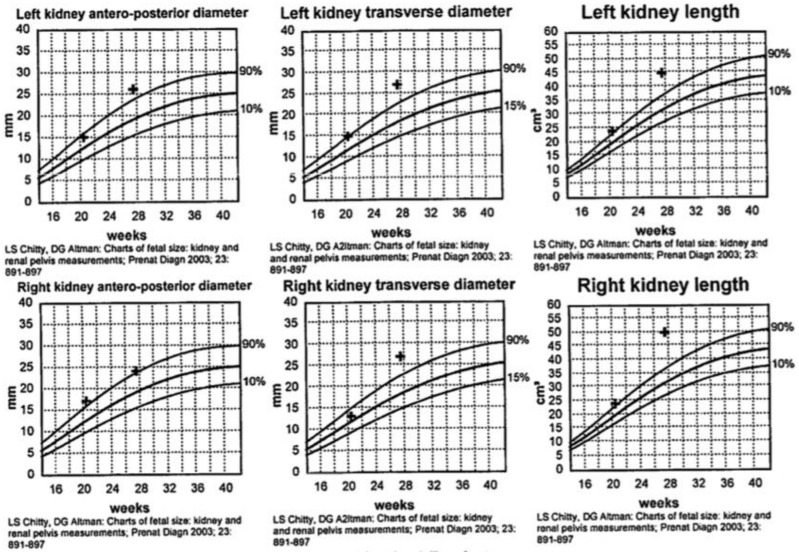
Growth curves of fetal kidneys from 21 to 27 weeks of gestation.

During her second trimester, the mother was noted to have progressively increasing edema, albeit with normal blood pressure. During a routine obstetrical visit at 27 weeks of gestation, she was noted to have 4+ urinary protein, and a 24 h urine collection revealed 11.58 grams of proteinuria, worsening shortly thereafter to 14.43 grams. Her creatinine at that time was 81 μmol/L. At the same visit, she had a fetal ultrasound, which revealed severe oligohydramnios with an Amniotic Fluid Index (AFI) of 1.7 cm, bilaterally enlarged echogenic kidneys (Figure 1), and an empty fetal bladder, albeit with a normal placenta and normal placental Doppler. The fetus was growing along the 50th centile. Figure 2 demonstrates the growth curves of the kidneys from 21 weeks to 27 weeks of gestation.

The couple was counseled regarding the differential diagnosis of neonatal renal failure. Microarray analysis was normal. Parental renal ultrasounds were normal, thus the testing strategy focused on autosomal recessive polycystic kidney disease (ARPKD). Sequencing of the related *PKHD1* gene showed no variant in the mother and a variant of unknown significance in the father (c.2162C>T (p.Thr721Met)). Maternal workup for glomerular-based disease included a normal serological screen. A kidney biopsy done at 30 weeks of gestation was consistent with focal segmental glomerulosclerosis (FSGS), including 17 glomeruli, none of which were globally sclerosed, but segmental sclerosis with capsular adhesions was noted in approximately 50% of the glomeruli. There was minimal interstitial fibrosis and tubular atrophy. Arteries and arterioles were normal. Immunofluorescence staining of scarred segments included only IgM and C3. Complete foot process effacement was noted.

**Figure 3:**
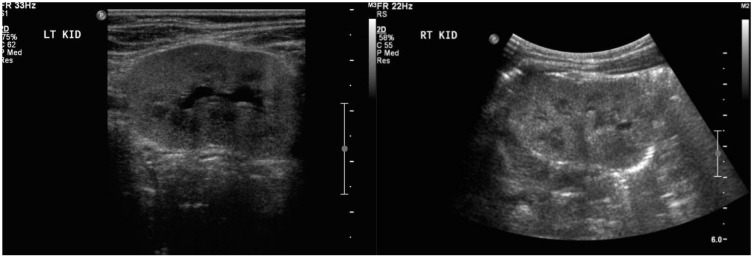
Ultrasound images of baby’s left and right kidney at 6 weeks of age.

Treatment with prednisone was introduced at 29 weeks of gestation. By 30 weeks of gestation, there was trace fluid within the fetal bladder, and by 33 weeks the AFI had normalized to 15 cm. However, the fetal kidneys remained echogenic until 35 weeks of gestation, then normalized, along with a normal AFI, Doppler examination, and biophysical profile. Growth improved to the 95th centile. Spontaneous vaginal delivery occurred at 37 weeks and 2 days of gestation. The birth weight was 3.045 kg (50–90th centile) and the APGAR scores were 9 and 9 at 1 and 5 min, respectively. Initial postnatal abdominal ultrasound showed mildly enlarged kidneys with diffuse increased parenchymal echogenicity, and urine analysis was abnormal for blood and increased protein (3 g/L), with a serum creatinine of 100 μmol/L. By 6 weeks postpartum, the baby had normal laboratory values for creatinine, albumin/creatinine ratio, and protein/creatinine ratio, and the urine dipstick was negative for proteinuria and blood. Abdominal ultrasound examination performed at this time showed that both kidneys had normal structure and were of age-appropriate size, with no detected abnormalities (Figure 3). The mother’s proteinuria improved on prednisone, but did not remit. Immunosuppressive therapy postpartum included tacrolimus, mycophenolate mofetil, and plasma exchange, after which she eventually entered into remission.

Serum for suPAR and the activation of its receptor integrin alphavbeta3 (AP5 activation) were assessed by established methods [11]. The measurement of serum suPAR was performed using a Human uPAR Quantikine ELISA kit (R&D Systems Inc., Minneapolis, MN, USA) following the manufacturer’s instructions. To semiquantitatively examine the effect of FSGS patient sera on podocyte β3 integrin activity, a human podocyte cell line (provided by Dr. Moin Salem, Bristol, UK) was cultured at 37 °C for 14 days for complete differentiation. Maternal serum demonstrated increased AP5 activation, while both elevated suPAR levels and AP5 activation were noted in the cord blood (Table 1), suggesting transplacental transmission of permeability factors that resulted in bilateral echogenic fetal kidneys, decreased fetal urine output, and, therefore, severe oligohydramnios.

**Figure 4:**
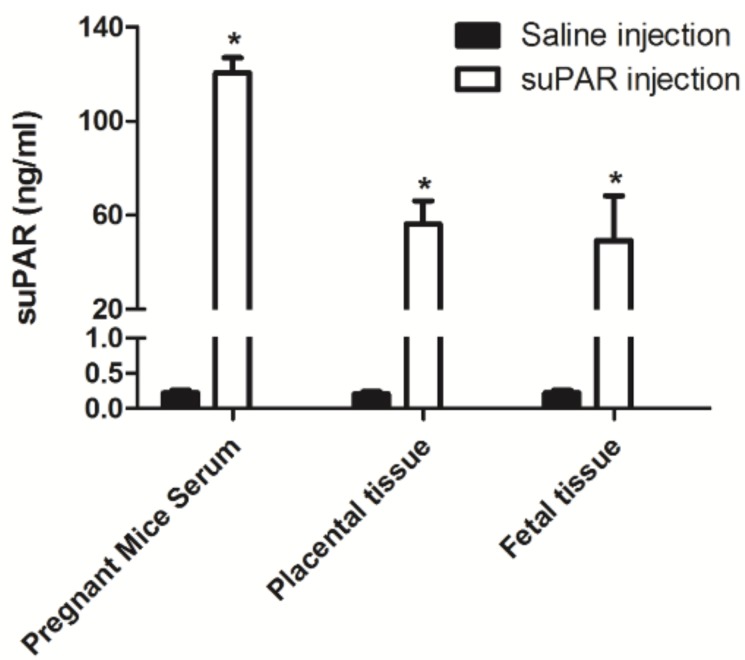
Maternal serum, placenta, and fetus tissue 2 hours after injection of suPAR.

In order to study the potential for suPAR to transfer from the mother to the fetus, we performed an in vivo study wherein 100 μL saline and human histadine (HIS)-tagged suPAR (20 μg) were injected in pregnant mice through their eyes (*N* = 2 for control saline injection and *N* = 4 for suPAR injection). Timed breeding was performed and injections were done on embryonic day 16.5. Maternal serum, placenta, and fetal tissue were collected 2 h after injection. Placenta and fetal tissues were lysed in Laemmli buffer for 20 min, then processed with homogenization and sonication (30 s on, 30 s off). suPAR level was determined by ELISA (ViroGates, Birkerød, Denmark). There was prominent transfer of suPAR from the mouse mother to the fetus (Figure 4). The substantially elevated human suPAR in pregnant mice demonstrated the success of injection. A significantly increased suPAR level in placental and fetal tissues indicated that suPAR was able to pass through the placental barrier to reach the fetus.

## 3. Discussion

FSGS is a common form of nephrotic syndrome, estimated to be responsible for 40% and 20% of adult and pediatric nephrotic syndromes, respectively [12]. In primary FSGS, it is thought that podocyte injury may be related to a circulating permeability factor. The evidence for a circulating factor is supported by the high recurrence rate of FSGS after kidney transplant (within hours to weeks) [13]. Serum urine-type plasminogen activator receptor (suPAR) has recently been identified as a potential circulating factor [6]. However, transplacental passage of maternal nephritogenic factors (the permeability factor in FSGS) to the fetus in the context of nephrotic syndrome has been rarely described.

Transplacental passage has been described in the context of membranous nephropathy. In 2002, Debiec et al. reported a case of transplacental transfer of antineutral endopeptidase antibodies in a mother who had a miscarriage 2 months before she became pregnant with the affected child [14]. She was found to have neutral endopeptidase deficiency, which caused alloimmunization at the time of the miscarriage and transplacental passage to the fetus. Prenatal ultrasound examination revealed oligohydramnios and enlarged fetal kidneys. No treatment was initiated during the pregnancy, and a male infant was born with oligo-/anuria, massive proteinuria, and respiratory distress on the first day of life. Low levels of circulating immune complexes were detected in the infant’s serum on day 13 of life, but no longer detected on day 40. Subsequently, at 11 months of age, the clinical examination was unremarkable. To the best of our knowledge, ours is the second case supporting transplacental passage of a maternal permeability factor to the fetus resulting in transient fetal and postnatal kidney dysfunction, but the first to document fetal echogenic kidneys and correction of the severe oligohydramnios with maternal treatment [9].

In our in vivo study, suPAR was injected and tracked in pregnant mice, suggesting that transmission occurred from mother to fetus. Although this finding should be confirmed by a larger study, it is consistent with the lower serum suPAR level noted in our pregnant patient at the time when the fetal content of suPAR was elevated to 4783 pg/mL in the cord blood, as presumably suPAR was being transferred from the mother to the fetus. Concomitantly, both the mother and the fetus displayed elevated integrin activation of podocytes when their sera were incubated with these cells. This finding is consistent with suPAR affecting the glomerular filtration barrier of both the mother and the fetus. Our case supports a permeability factor, such as suPAR, as a potential underlying cause of podocyte injury in primary FSGS and sheds light on the effect of this factor on the fetus. The improvement in the amniotic fluid volume was likely related to the maternal treatment with steroids. The resolution of the newborn’s proteinuria was likely related to complete elimination of the permeability factor by her otherwise normal kidneys.

Our case also expands the differential diagnosis of fetal echogenic kidneys to include maternal nephrotic syndrome. Fetal renal echogenicity otherwise is a heterogeneous condition with a broad differential diagnosis, including intrinsic renal diseases such as autosomal dominant and autosomal recessive polycystic kidney disease, aneuploidy (mainly trisomy 13 and 18), infections such as cytomegalovirus, overgrowth syndromes such as Beckwith-Wiedemann syndrome, Perlman and Simpson-Golabi-Behmel syndrome, renal vein thrombosis, and congenital nephrotic syndrome, as well as secondary to obstructive uropathy [15,16]. There has not been a reported association between this prenatal ultrasound finding and maternal nephrotic syndrome apart from the previously reported case of membranous nephropathy [14].

In conclusion, our case contributes to further expanding our knowledge about the association between maternal nephrotic syndrome and fetal kidney manifestations, including renal echogenicity. Further, our case is the first to demonstrate prenatal improvement of the fetal kidney manifestations following maternal therapy, providing important information for the management of similarly affected pregnancies. Finally, the case further supports the role of circulating permeability factors as an underlying mechanism in some cases of primary FSGS.

## Figures and Tables

**Table 1:** Soluble urokinase-type plasminogen activator receptor (SuPAR) and AP5 activity at different time points in gestation.

	SuPAR (pg/mL)	AP5	Treatment	Urine Protein (g/day)
30 weeks of gestation	1821	1.78	Prednisone 50 mg	9.77
34 weeks of gestation	1998	1.54	Prednisone 50 mg	14.4
Delivery cord blood	4783	1.61		
